# Metabolic Checkpoints in Differentiation of Helper T Cells in Tissue Inflammation

**DOI:** 10.3389/fimmu.2018.03036

**Published:** 2019-01-14

**Authors:** Suyasha Roy, Zaigham Abbas Rizvi, Amit Awasthi

**Affiliations:** Immuno-Biology Lab, Translational Health Science and Technology Institute, Faridabad, India

**Keywords:** T cell, cytokines, inflammation, metabolism, checkpoint, transcription factor

## Abstract

Naïve CD4^+^ T cell differentiate into effector and regulatory subsets of helper T (Th) cells in various pathophysiological conditions and modulate tissue inflammation in autoimmune diseases. While cytokines play a key role in determining the fate of Th cells differentiation, metabolites, and metabolic pathways profoundly influence Th cells fate and their functions. Emerging literature suggests that interplay between metabolic pathways and cytokines potentiates T cell differentiation and functions in tissue inflammation in autoimmune diseases. Metabolic pathways, which are essential for the differentiation and functions of Th cell subsets, are regulated by cytokines, nutrients, growth factors, local oxygen levels, co-activation receptors, and metabolites. Dysregulation of metabolic pathways not only alters metabolic regulators in Th cells but also affect the outcome of tissue inflammation in autoimmune and allergic diseases. Understanding the modulation of metabolic pathways during T cells differentiation may potentially lead to a therapeutic strategy for immune-modulation of autoimmune and allergic diseases. In this review, we summarize the role of metabolic checkpoints and their crosstalk with different master transcription factors and signaling molecules in differentiation and function of Th subsets, which may potentially unravel novel therapeutic interventions for tissue inflammation and autoimmune disorders.

## Introduction

**Figure 1:**
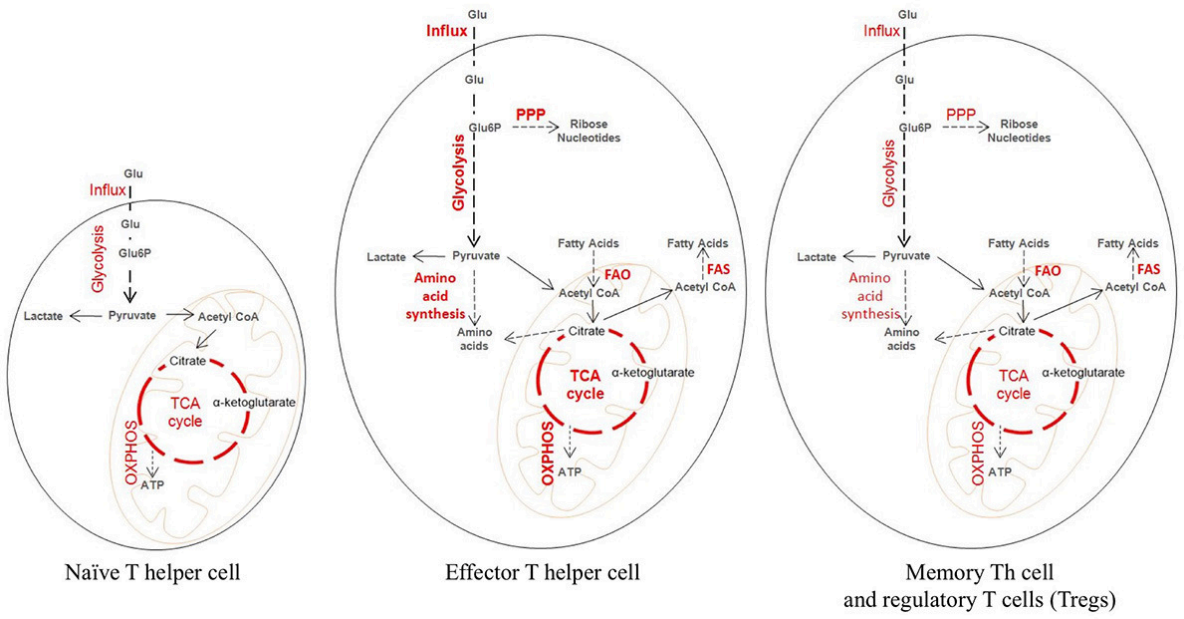
Metabolic state of naïve and activated T cells during tissue inflammation. Metabolically a naïve T cells behave like a resting cell during homeostasis. Glucose is broken down at steady rate into pyruvate which then enters the tri-carboxylic acid (TCA) cycle leading to the generation of ATP needed for cellular energy. Upon activation, a naïve T cell differentiate into an effector and a memory T cell, which differ considerably from the naïve T cell in their metabolic profile. Effector T cells are characterized by increased glucose transporter 1 (GLUT 1) expression needed for higher influx of Glucose. The influxed glucose is then catabolized through increased rate of glycolysis. The intermediate products of glycolysis are utilized for the generation of biosynthetic precursor through pentose phosphate pathway (PPP) and amino acid synthesis. Also, there is increased fatty acid oxidation (FAO) and in some cases increased Fatty acid synthesis (FAS). The memory T cells and the Tregs behave similar to the naïve T cells metabolically. They maintain a steady rate of glycolysis, TCA cycle, and oxidative phosphorylation (OXPHOS) producing ATP. The striking characteristic of memory T and Treg cells is high FAO and FAS.

Nutrients, water, and oxygen are the fundamental constituents that are required for all the living cells, even more so for the cells of the immune system, which are metabolically hyper active during immunological reactions (1). In general, the energy requirement of resting naïve T cells is fulfilled by aerobic metabolism of glucose via oxidative phosphorylation. However, upon antigenic stimulation, naïve T cells get activated, and metabolically shift toward aerobic glycolysis (1, 2). The nutrients in the form of metabolites are not only essential for maintaining T cell homeostasis but are also essential for generating precursors of bio-molecules, which support rapid proliferation of activated T cells essential for their functions. The metabolic changes occurring within T cells influence the fate of diverse immune responses during infection, inflammation, and autoimmunity (Figure 1). In order to control hyper activation of T cells in an immune reaction, various regulatory mechanisms have been identified. Metabolic checkpoints were identified as one of the key regulator of T cell responses (3). Classically, the activity of metabolic enzyme or concentration of a specific metabolite was suggested to be important checkpoint in immune response in infection and inflammation in autoimmunity. However, emerging literature indicates that metabolic checkpoint could be an enzyme, metabolite, a signaling molecule, and/or even a transcription factor that could potentially regulate T cells differentiation and functions (Figure 1).

Metabolism is a dynamic process, which provides essential building blocks for diverse cellular processes and fulfills energy requirements of cells. In addition to maintaining essential functions of cells, metabolites regulate cellular differentiation, and functions (4). The metabolic reprogramming of an activated T cell is essential for its rapid proliferation and acquisition of effector functions (2). Published literature have clearly indicated the distinct metabolic requirements of undifferentiated naïve vs. activated and differentiated T cells, as undifferentiated naïve T cells predominantly rely upon ATP produced via oxidative phosphorylation and β-oxidation of fatty acids while activated/differentiated T cells met their energy demands primarily by glycolytic, pentose-phosphate, and glutaminolysis pathways (5).

**Figure 2:**
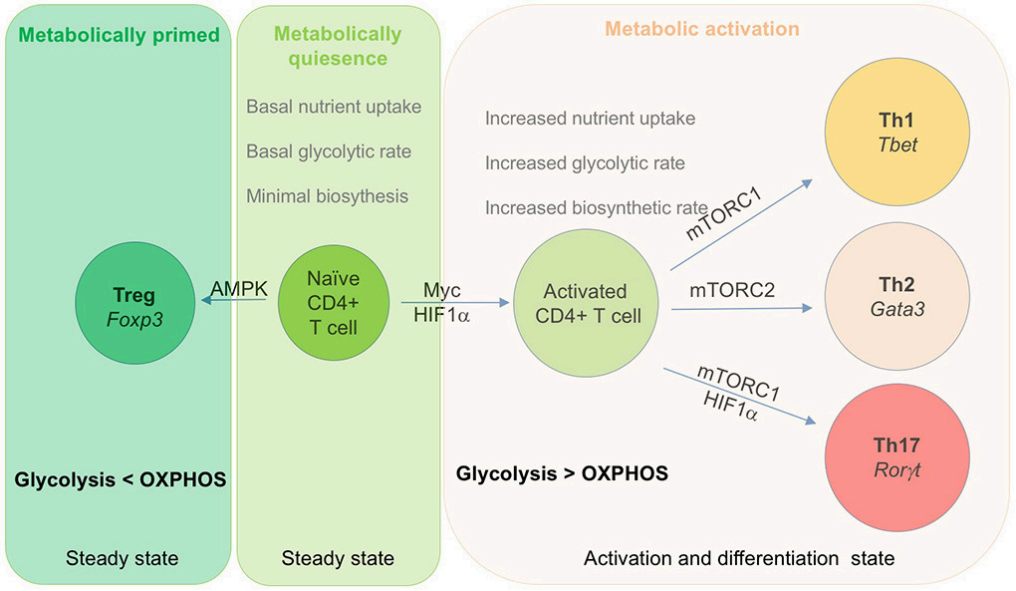
Role of metabolic checkpoints transcription factor in T cells activation and differentiation. Metabolism play a key role in activation and differentiation of effector subset of T cells. This diagram depict key transcription factor that regulate the differentiation of effector cells. Naïve T cells are metabolically quiescent and rely on OXPHOS for their energy requirement. Upon activation, T cells shift their metabolic requirements and utilizes glycolysis over OXPHOS to meet their growing energy and biosynthetic demands. Both Myc and HIF-1α play a crucial role in activation of T cells upon antigen encounter. During this process, activated T cells express Glut 1, a glucose transporter that enhance glucose uptake in order to maintain the biosynthetic demand of dividing cells. Activated T cells differentiate into Th1 and Th2 cells by respectively expressing T-bet and Gata3 as their unique master transcription factor. In addition, Th1 and Th2 cells also express mTORC1 and mTORC2 as metabolic transcription factor respectively. Similarly, Th17 cells express RORγt as its lineage specific transcription factor along with HIF-1α and mTORC1.

Activated CD4^+^ T cells proliferate and acquire distinct effector phenotypes such as Th1, Th2, Th9, and Th17 cells, which contribute to specialized functions in eliminating intra and extracellular pathogens as well as inducing tissue inflammation in autoimmunity and allergic inflammation (6). On contrary, regulatory subsets of CD4^+^ T cells, which include Foxp3^+^ regulatory T cells (Tregs) and type 1 regulatory T (Tr1) cells, suppress effector T cell functions and contribute to resolution of tissue inflammation in autoimmune diseases (6) (Figure 2). Each of these effector and regulatory subsets of T cells differentiate in the presence of specific differentiating cytokines, cell signaling modules, and unique sets of transcriptional network which regulates the distinct metabolic reprogramming in both the effector and regulatory T cells. For example, Foxp3^+^ Tregs, and memory T cells primarily rely on lipid oxidation while effector T cells utilizes glycolytic and glutaminolytic pathway to support their survival (6). In this review, we discuss and summarize important metabolic checkpoints of T helper cell differentiation and function in immunity and autoimmunity. We further describe the current advances as to how metabolic reprogramming of T cells regulates their effector functions in an immune response.

## Metabolites and Metabolic Signals Acts as a Checkpoint in T Helper Cell Differentiation

Emerging data in cancer biology has established that specific metabolites may act as signaling components of specific metabolic pathways, which affect the physiology of the cancer cells. Similar to cancer cells, T cells, upon antigenic stimulation, rapidly proliferate, suggesting a possibility of involvement of metabolites in T cells activation and proliferation. The metabolic components, ranging from metabolites, enzyme complexes to signaling molecules, regulate T cell activation, differentiation, and functions in various patho-physiological conditions. Many of these metabolites could act as checkpoints during Th cell differentiation in tissue inflammation. Here we discuss some of the examples as to how metabolites influence T cells fate and their effector functions.

### Glucose

Glucose, the primary source of cellular energy, feeds into pathways that generate metabolites essential for cellular growth, and functions. Glucose provides instant energy for activated and proliferating T cells as well as metabolic precursors such as nucleic acids, proteins, lipids, and carbohydrates. The increased energy demand of rapidly dividing cancer cells is fulfilled by the upregulation of glycolysis (7), and this phenomenon, which was initially described in cancer cells, is known as “Warburg Effect” (8). Similar to tumor cells, rapidly proliferating activated T cells also rely on aerobic glycolysis, instead of oxidative phosphorylation, to support their cellular energy requirements as well as biosynthetic metabolic demands (8). A differentiating pro-inflammatory T cells shift to aerobic glycolysis, which lead to lactate production even in the abundance of oxygen (8, 9). The “Warburg Effect” in T cells begins, with the engagement of T cell receptor (TCR) with its ligand that leads to T cells activation, and triggers rapid glucose uptake via upregulation of glucose transporter, Glut1 (8, 9). In addition to TCR activation and co-stimulatory signals of CD28, which activate PI3K/AKT-mediated translocation of Glut1 from the intracellular vesicles to the cell surface of activated T cells (10). Upon translocation through its transporter, glucose is catabolized to provide the building blocks for cellular proliferation and generate energy via TCA cycle, OxPhos, or Warburg metabolism.

The critical function of glucose in T cell activation and differentiation was identified in a study where deprivation of glucose leads to impairment of T cell activation and survival (10). Interestingly, T cell survival and functions could not be rescued by an alternate energy source to glucose such as glutamine (10, 11). In addition to T cell survival, the effect of glucose deprivation was also found to be associated with cytokines production by T cells, as the absence of glucose in T cell cultures results in decreased IFN-γ production (10, 12). On contrary, transgenic expression of Glut1 in T cells induces enhanced production of IL-2 and IFN-γ, suggesting a critical role of glucose in T cell activation and functions (12). Consistently, it was demonstrated that the deficiency of Glut1 profoundly suppressed glucose metabolism that lead to a decrease in T cells differentiation and functions (12). Interestingly, in contrast to effector T cell subsets, Tregs functions remain intact in Glut1 deficiency (12). Taken together, these observations indicate that glucose metabolism is essential for effector T cell functions but not for Tregs functions. The role of glucose and its metabolism has also been implicated in T cell dependent function of B cell activation and antibody production (13).

Activated T cells prefer glycolysis over OxPhos despite glycolysis provides lesser ATP than Oxphos (14). The advantage of glycolysis over Oxphos is to provide biosynthetic precursors that are essential for the generation of amino acids, lipids and nucleic acids for rapidly proliferating T cells. The rate of glycolysis in T cell activation determine the outcome of the generation of effector and memory T cells repertoire. Further, it is known that metabolic status of Tr1 is similar to the effector T cells, however Tregs are known to adopt low glycolysis and high oxidative metabolism (14–16). Effector T cell populations such as Th1, Th2, Th9, and Th17 were shown to have enhanced glycolytic activity while memory T cells and Tregs rely primarily on fatty acid oxidation for their survival (5, 14, 15, 17). Consistently, blocking glycolysis using 2-DG, a prototypical inhibitor of hexokinase (first rate-limiting enzyme of glycolysis), was found to suppress the differentiation of both CD4^+^ and CD8^+^ T cells into their effector subsets, indicating the importance of glucose metabolism and glycolysis in T cells activation and functions (18). Mechanistically, glucose metabolism is primarily regulated by PI3K/AKT/mTOR pathway, signal transducer and activator of transcription 5 (STAT5), extracellular signal-related kinase (ERK) and Mitogen-Activated Protein Kinases (MAPKs) (19, 20). Consistently, high glucose level or hyperglycemia has long been associated with the onset of chronic inflammatory diseases such as rheumatoid arthritis (RA), multiple sclerosis (MS), as proteomic analysis of synovial fluid has identified the proteins critical for glycolytic pathways are overexpressed in RA patients (21). This observation is in accordance with upregulation of glycolytic flux in synovial lesions. In patients with such conditions, glucocorticoid therapy has helped conventionally to overcome inflammatory conditions associated with inflammatory T cells (21). In addition, recent studies with exogenous insulin treatment have also shown promising result in lowering the glucose level to normal (22).

### Lipids

Fatty acids composition, particularly of the cell membrane, regulates variety of physiological processes including cellular signaling, which ultimately leads to modulation of inflammatory response and T cells functions in autoimmune diseases (23). The role of glucose and glutamine in sustaining T cells survival and effector functions is well-understood, however little is known about fatty acids metabolism and their effect on regulating T cell differentiation and functions. Dietary fatty acids, omega 6, and omega 3 polyunsaturated fatty acids (PUFAs), are known to regulate inflammatory conditions, as omega 3 PUFAs act as anti-inflammatory mediators in inflammatory diseases such as psoriasis, RA, and IBD in mouse model of TNBS-induced ulcerative colitis (24).

Fatty acid metabolism remains an integral part of T cell differentiation in tissue inflammation, as T cell activation induces cellular lipid biosynthetic pathways that are linked to glucose metabolism (25). β-oxidation of fatty acids is essential for the generation and functions of Foxp3^+^ iTregs, and is required for the membrane synthesis of proliferating effector T cell populations such as Th1, Th2, Th9, and Th17 cells (17, 26). Proliferating activated T cells augment fatty acid synthesis with a concomitant reduction in fatty acid oxidation. Fatty acid synthesis and fatty acid oxidation occur in cytosol and mitochondria, respectively. While fatty acid synthesis requires ATP consumption, fatty acid oxidation generates ATP via acetyl-CoA-TCA-OXPHOS cycle.

Fatty acids metabolic pathways are crucial in the interplay between Th17 and Foxp3^+^ Tregs functions and their generations (26). The generation of Th17 and Foxp3^+^ Tregs are reciprocally regulated, as TGF-β1 induces the generation of Foxp3^+^ Tregs cell while IL-6 together with TGF-β1 not only suppresses the generation of Tregs but induces the differentiation of Th17 cells (27). It was demonstrated that Fatty acids contribute in generation and functions of Tregs cells while their intracellular accumulation was found to modulate the pathogenicity of Th17 cells.

Acetyl-CoA carboxylase 1 (ACC1) plays a critical role in fatty acid synthesis by converting acetyl-CoA to malonyl-CoA (26). ACC1-mediated fatty acid synthesis is crucial for the generation of Th17 cells, as genetic deficiency of ACC1 leads to defective Th17 cell response (26). In contrast, ACC1 deficiency induces the generation of Foxp3^+^ Tregs cells (26). Consistent with these findings, adoptive transfer of ACC1-deficient T cells in mouse model of GVHD reduced mortality associated with enhanced frequency of Foxp3^+^ Tregs cells, suggesting that fatty acid regulates effector T cells while enhances Tregs functions. Furthermore, oxidation of fatty acid is essential for the generation of iTregs via upregulation of AMPK, which is further shown to phosphorylate ACC, the key enzyme for fatty acid synthesis (17, 26, 28, 29). AMPK inactivates both the isozymes, ACC1 and ACC2, and thus downregulates fatty acid synthesis. Moreover, AMPK upregulates the expression of carnitine palmitoyltransferase I (CPT1), the rate-limiting enzyme in fatty acid oxidation and promoting the differentiation of Tregs. Consistently, mice deficient in AMPK were shown to have severe tissue inflammation due to an impaired development and functions of Tregs, which in turn induces exaggerated differentiation of effector T cell populations such as Th1, Th2, Th17 (17, 26, 28, 29).

Using the single cell RNA sequencing, the role of fatty acid in the pathogenicity of Th17 cells was further identified (30). The expression of CD5L (CD5 antigen-like) in Th17 cells regulate the pathogenic functions of Th17 cells by modulating the functions of RORγt, a master transcription factor of Th17 cells (30). The functions of CD5L was found to be associated with lipid metabolism, as it suppresses fatty acid synthase (30). It was demonstrated that CD5L promotes the binding of RORγt to *Il10* gene locus while suppressing RORγt binding to *Il23r* and *Il17* gene locus in Th17 cells, thus enhancing the development of non-pathogenic Th17 cells (30).

Factors that affect lipid synthesis were also found to be associated in regulation of interplay between effector and regulatory T cells in tissue inflammation. Lipid synthesis was shown to be regulated by transcription factor Myc, as Myc-deficient cells was found to have lower levels of lipid synthesis, which leads to reciprocal regulation of effector and regulatory T cells in tissue inflammation (25). In addition, cell signaling kinases such as mTOR are also critical for lipid biosynthesis, as inhibition of mTOR using rapamycin substantially reduced fatty acid synthesis upon T cell activation due to impairment of Myc induction (25). Upon T cell activation, PI3K and mTOR induces the expression of sterol regulatory element-binding proteins (SREBPs), which bind to the promoter of fatty acid synthesis (FAS)-specific genes (31). Taken together, the role of fatty acid is clearly implicated in regulation of tissue inflammation by enhancing the generation and functions of Tregs.

In addition to fatty acids, cholesterol, an essential component of cellular membranes, is required for T-cell activation and proliferation (32). It was suggested that an increase in cellular cholesterol helps in fighting bacterial infection by promoting inflammation (32, 33). However, in chronic metabolic inflammatory conditions such as obesity and atherosclerosis hypercholesterolemia, cholesterol is known to worsen the disease conditions (33). Similarly, an increased level of cholesterol was found in sera samples of RA patients, suggesting a pathogenic role of cholesterol in promoting tissue inflammation in RA (34–36). In autoimmune diseases like RA and systemic lupus erythematosus (SLE), a disturbed cholesterol efflux homeostasis results in worsening of the disease, and such patients were shown to have therapeutics effects by administration of high-density lipoproteins (37, 38). Cholesterol promotes the activation, differentiation, and proliferation of both CD4^+^ and CD8^+^ T cells via suppression of LXRβ and activation of sterol response-element-binding protein-2 (SREBP2) (39). Furthermore, SREBP2 increases cholesterol synthesis, activating PI3K-mTOR pathway, which is crucial for T cell activation and differentiation; while LXRβ inhibits the cholesterol deposition thereby suppressing the T cell activation and proliferation (31, 39). Molecularly, cholesterol regulates TCR signaling by binding to the TCRβ chain, enhancing its avidity for MHC-Peptide complex through the formation of membrane raft (32). It has been recently reported that accumulation of intracellular cholesterol through mevalonic acid pathway drives Th17 cell differentiation (40). Interestingly, oxysterols such as 7α,27-OHC and 7β,27-OHC acts as RORγt agonists that binds to ligand binding domain of RORγt further activate its binding to *Il17* gene locus as well as other Th17 cells promoting factors to potentiate Th17 cell differentiation (40). Moreover, LXR inhibits Th17 cell differentiation by interfering with the aryl hydrocarbon receptor mediated IL-17 transcription (41). Blocking of mevalonate pathway for cholesterol biosynthesis by atorvastatin inhibits Th1 cell differentiation and pro-inflammatory response during experimental autoimmune encephalomyelitis (EAE) (42).

### Nitric Oxide

Nitric oxide (NO) is a highly reactive free radical, which plays an important role in mediating numerous biological functions such as vasodilation, platelet aggregation, smooth muscle cell proliferation, superoxide radical generation, monocyte adhesion, LDL oxidation, and immune regulation etc. Briefly, NO is derived from L-Arginine in a reaction catalyzed by nitric oxide synthase (NOS). There are three different forms of NOS: neuronal nitric oxide synthase (nNOS or NOS1), inducible nitric oxide synthase (iNOS or NOS2), and endothelial nitric oxide synthase (eNOS or NOS3). Nitric oxide production in immune cells is primarily regulated by inducible NOS or iNOS, which is activated by different immunological stimuli such as lipopolysaccharide (LPS), interferon-γ (IFN-γ), tumor necrosis factor α (TNF-α), interleukin 1β (IL-1β) generated during immune response (43–45).

The level of NO acts as a disease index in inflammation in many diseases. For example, in RA or osteoarthritis patients, increased levels of NO were found in the synovial fluid and serum of the inflamed joints, suggesting an association of NO with disease pathogenesis (46–48). Similarly, non-steroidal anti-inflammatory drugs (NSAIDs) are helpful in treating high levels of excretory urinary nitrate (an indicator of NO in kidney during arthritis) (49, 50).

In addition, NO also acts as a mediator during tissue inflammation in IBD, as production of NO was found to be increased in IBD patients, and therefore found to be associated with the enhanced activity of iNOS in the inflamed mucosa of the gut (51). The primary source of enhanced NO in IBD patients was monocytes, lymphocytes, macrophages, and neutrophils, and these patients were characterized by enhanced urinary nitrate excretion (51, 52). Moreover, treatment with NSAIDs is found to have similar reducing results for nitrate excretion as in RA. Based on the clinical observations, iNOS has become one of the most prominent therapeutic target for IBD (52).

Since the role of NO was well-established in inflammatory conditions of arthritis and IBD, therefore it is essential to understand its influence on effector functions and differentiation of T cell subsets. Similar to cytokines, NO is a soluble factor that was shown to influence the differentiation of Th cell. The expression of iNOS is induced in activated CD4^+^ T cells through distinct signaling pathways triggered by micro-environmental cues in the extracellular milieu. The role of NO was extensively studied in reciprocal regulation of Th17 and Tregs cells, as NO negatively regulates Th17 cell differentiation by nitrating the tyrosine residues of RORγt, which leads to inhibition of the transcriptional activity of RORγt in Th17 cells (53). Consistently, iNOS deficient mice were found to be more susceptible for EAE and experimental colitis associated with accumulation of higher frequency of Th17 cells in their target tissues (53). Contrary to iNOS deficiency, increased NO using NO donor, NOC-18, found to inhibit the development of Th17 cells by suppressing aryl hydrocarbon receptor (AHR) expression, and thus inhibiting Th17 cell differentiation (54). In addition to blocking RORγt functions, NO also found to block Th17 cell differentiation by antagonizing the functions of IL-6, as IL-6 is one of the key differentiating factor that induces *de novo* differentiation of Th17 cells (55).

NO modulates the differentiation of Th1, Th17, and Tregs by modulating their respective differentiating cytokines such as IL-12, IL-6, and TGF-β1. NO, together with IL-6, was found to potentiate the suppression of Tregs development induced by TGF-β1 and retinoic acid, as retinoic acid is known to enhance Foxp3 expression in the presence of TGF-β1, therefore stabilizing the functions of Tregs (55). It was suggested that higher amounts of retinoic acid overcome the repression of Foxp3 induced by NO and IL-6 and leads to the predominance of Foxp3^+^ Tregs over Th1/Th17 cells (55). Although much of the published literature suggested that NO suppresses the differentiation of Th17 cells, it has also been shown that NO is critically required for the induction and stability of human Th17 cells (56). Physiological concentration of NO was shown to promote the generation of Th17 cells (56), as blocking of NOS2, or cGMP–cGK signaling pathway suppresses the *de novo* induction of Th17 cells in ovarian cancer patients (56).

In addition to Th17 cells, the effect of NO was shown to modulate TGF-β1 activity in potentiating Th1 cell differentiation in both IL-12-dependent and -independent manner (55). Although NO potentiates Th1 cell differentiation induced by IL-12 in an inflammatory environment, it was also suggested that NO maintains Th1 response even in the absence of IL-12 (57). NO was shown to enhance IFN-γ-dependent expression of T-bet, which further promote Th1 differentiation even in the absence of IL-12 (57). Even though the effect of TGF-β1 is dominant on IFN-γ signaling that result in the shift from Th1 to iTregs, however presence of NO potentiates STAT1-mediated IFN-γ signaling, inhibiting TGF-β1-induced Foxp3 expression, and thereby reinforces Th1 development (57).

In addition to Th1 and Th17 cells, the effect of NO was also shown on the development of Th9 cells. It was demonstrated that NO enhances differentiation of Th9 cell, and therefore exacerbates IL-9, and Th9 dependent allergic inflammation in asthma (58). Mechanistically, NO was shown to enhance both TGF-β1, and IL-4 signaling pathways to promote the development of Th9 cells. Moreover, NO nitrosylates MDM2 at cysteine residue thereby derepressing p53 from MDM2-p53 complex, which induce IL-2 production and activate STAT5-IRF4 axis to promote Th9 differentiation (58). In addition, NO was found to increase the surface expression of IL-4R, which potentiates STAT6-mediated IRF4 dependent expression of IL-9 in Th9 cells (58). Other than IL-4-IL-4R signaling, NO was also shown to enhance TGFβR expression, which in turn enhances TGF-β1 dependent binding of PU.1 to *Il9* promoter in differentiation of Th9 cells (58). Consistently with these observations, NO was found to exacerbate airway inflammation while *iNOS*^−/−^ mice were shown to have attenuated airway inflammation due to a decreased frequency of Th9 cells (58). Taken together, these observations clearly indicated that NO affect helper T cells differentiation and functions in autoimmune as well as allergic inflammation, and therefore modulating NO could be a potential metabolic checkpoint in T cells differentiation and disease pathophysiology.

### Adenosine Triphosphate (ATP)

During the metabolic process, adenosine triphosphate (ATP) is generated, and used primarily as a source of energy for cells. In addition to energy source, ATP can act as an extracellular signaling molecule, which mediate cell to cell communication in an autocrine and paracrine manner (59, 60). Glucose feeds into glycolysis to generate ATP during T cell activation and proliferation. In this process of glycolysis, one glucose molecule is metabolized to provide two reduced nicotinamide adenine dinucleotide (NADPH), two molecules of ATP and two pyruvate molecules. Pyruvate further feeds into tricarboxylic acid cycle (TCA) in less active cells such as memory cells and Foxp3^+^ Tregs. In TCA cycle, pyruvate is metabolized to generate NADH and reduced flavin adenine dinucleotide (FADH2), which further feeds into OXPHOS, an oxygen dependent step, and generate 36 ATP molecules for one molecule of glucose.

Under physiological conditions, the cellular ATP can be released from healthy cells through exocytosis while necrotic or apoptotic cells releases ATP under pathological conditions (61). As a messenger, extracellular ATP binds to purinergic receptors, P2X, and P2Y that are present on the cell surface. Upon T cell activation via TCR, ATP contributes to the activation of MAPK signaling cascade through P2X receptor and contribute to T cell activation (62). The binding of ATP to its receptors initiates a signaling cascade and integrate various cellular signaling events in inflammatory conditions in both human and mouse T cells (63, 64). Studies have shown that extracellular ATP triggers the effector CD4^+^ T cells proliferation while inhibiting the functions of Tregs (65). ATP was found to enhance the differentiation of Th17 cells and exacerbated T-cell-mediated colitis in mouse model (66). Mechanistically, ATP increases the number of CD70^high^CD11c^low^ lamina propria cells in germ-free mice and upregulate the expression of TGF-β1, IL-6, IL-23p19, which together induce Th17 cell differentiation (66). IL-6, which support the generation of Th17 cells, enhances the generation of cellular ATP as well to further enhance the generation of Th17 cells in a feed forward loop (66). It was further demonstrated that IL-6-mediated ATP-P2XY signaling converts Foxp3^+^ Tregs into Th17 cells *in vivo*, suggesting that ATP can control Tregs and Th17 cells reciprocally (62). In addition to Th17 cells, the role of extracellular ATP was also tested in generation and functions of Foxp3^+^ Tregs, as it was demonstrated that P2XY receptor is highly enriched on Foxp3^+^ Tregs. Consistently, extracellular ATP was found to suppress the stability and functions of Foxp3^+^ Tregs (62). Mechanistically, it was shown that Foxp3^+^ Tregs have a specialized function that control extracellular ATP by breaking it down to AMP via CD39, a nucleoside triphosphate diphosphohydrolase-1 (NTPDase 1). The surface expression of CD39 was found to be highly enriched on Foxp3^+^ Tregs cells, and is driven by Foxp3 (67). These observations thus imply that Tregs have an intrinsic capacity to control effector cell functions in tissue inflammation by limiting ATP concentration in inflammatory milieu.

Since extracellular ATP provides pro-inflammatory signal to effector T cells and also helps in maturation of dendritic cells, therefore controlling the presence of ATP might be a potential strategy for suppressing tissue inflammations.

Other than Foxp3^+^ Tregs, extracellular ATP inhibits Tr1 differentiation by triggering the inactivation of AHR, which is crucial for Tr1 cell differentiation and functions (16). Molecularly, it was demonstrated that ATP induces the expression of HIF-1α, which bind and inhibit the activation of AHR and ARNT (AHR nuclear translocator) thereby blocking Tr1 cell differentiation (16). Moreover, ATP interferes in recruitment of AHR to *Il10, Il21*, and *Entpd1* promoters, and therefore repressing their expression in Tr1 cells (16). Altogether it suggests that extracellular ATP provide crucial signal in maintaining the balance between effector and regulatory T cells in physiological and disease conditions, and therefore ATP could be a potentially metabolic checkpoint to modulate effector and regulatory T cells functions in tissue inflammation.

### Nicotinamide Adenine Dinucleotide (NAD)

NAD is another important energy metabolite which is released by cells through lysis during cellular damage or inflammation mediate intracellular signaling. It has been reported that NAD promotes Th1, Th2, and Th17 cells differentiation (68). Genome-wide transcriptomics analysis identified the upregulation of tryptophan hydroxylase-1 (*Tph1)* in Th1, Th2, and iTreg cells in the presence of NAD which facilitates their differentiation (68). Moreover, it was found that NAD triggers IL-10 production in Th1 cells and IL-10 and IL-17 production in Th2 cells via upregulation of *Tph1*, which provide protection against EAE by promoting remyelination and axonal regeneration. In addition, NAD skews iTreg cells into Th17 cells, which produce TGF-β1 and have immunosuppressive properties (68).

NAD not only induces immunosuppressive T cells, but also inhibits T cell proliferation via activation of P2X7 receptor signaling (69). Mechanistically, NAD leads to ART2.2-mediated ADP-ribosylation of the cytolytic P2X7 receptor expressed on T cells. Tregs express mono–ADP-ribosyltransferase (ART2.2) which catalyzes the covalent transfer of the ADP-ribose group from NAD^+^ onto arginine residues of membrane target proteins resulting in NAD induced cell death of Treg cells (70, 71). The deleterious effects of NAD on Treg cells can be protected by an inhibitory ART2.2-specific single domain antibody (72). In tumor mouse models, administration of exogeneous NAD induces antitumor immune response by selective depletion of Treg cells (72). Thus, NAD is one of the major metabolic signals which serve as checkpoint for CD4^+^ T cell differentiation with anti-tumor properties and can be used to treat autoimmune diseases such as MS, IBD and chronic and inflammatory diseases.

## Transcription Factors as Metabolic Checkpoints of T Cell Differentiation

The interplay between different signaling cascades together play a vital role in regulating T cell differentiation. A number of transcription factors have been reported that regulate different metabolic pathways crucial for T cell differentiation and functions. Here we discussed the role of transcription factors that act as metabolic checkpoints in Th cells differentiation and functions.

### Myc

Myc, a basic helix–loop–helix leucine zipper transcription factor, is induced upon TCR signaling and essential in metabolic reprogramming of T cells activation and functions (73). Expression of Myc is essential for glycolysis and glutaminolysis in activated T cells, as Myc induces the upregulation of GLUT1; also, known as SLC2A1, which is required for glucose uptake as well as functions of pyruvate kinase, lactate dehydrogenase A (LDHA) and hexokinase in glycolysis (73). In addition, it was suggested that Myc promotes glutaminolysis by increasing the expression of glutaminase and glutamine transporters (5). Glycolysis and glutaminolysis are essential for proliferating effector Th cell populations like Th1, Th2 and Th17 cells, therefore the role of Myc in generation of effector T cell was proposed (5). Although Myc and HIF-1α are two most critical transcription factors essential for metabolically active effector T cells, it was found that Myc, but not HIF-1α, is responsible for metabolic reprogramming in T cell activation and rapid cell divisions in proliferating T cells (73).

The role of Myc was identified in regulation of effector and regulatory T cells generation and functions, as Myc found to be essential in maintaining the balance between Th17/Foxp3^+^ Treg cells differentiation. Gomez-Rodriguez et al have shown that Myc deficiency in the Itk^−/−^ leads to impaired Th17 cell differentiation due to increased expression of Pten (74). In addition, Myc activates miR-19b, which is found to repress PTEN and enhance STAT5 activation. Myc is also found to activate IL-2-mediated PI3K-mTOR pathway that results in enhanced Th17 cells differentiation (74). Deletion of Myc in CD4^+^ T cells inhibits PI3K-mTOR pathway, which lead to an increased expression of Foxp3 and resulted in enhanced frequency of Tregs cells. Based on the emerging literature, it is clearly evident that metabolically active T cells require Myc activity, which is essential for modulation of effector and regulatory T cells generation and functions.

### HIF-1α

In addition to Myc, metabolically active T cells express increased levels of HIF-1α. Transcription factor HIF-1α forms a heterodimer and composed of two subunits, and is induced in response to low oxygen concentrations in a state of hypoxia. Under hypoxic condition, HIF-1α dimerizes with HIF-1β, a constitutively expressed ARNT, and translocate into the nucleus where it binds to hypoxia responsive elements (HREs) and mediate transcription of its target genes (75). Under the optimal oxygen supply, a state called normoxia, HIF-1α gets hydroxylated at proline residues by prolyl hydroxylases, which make HIF-1α sensitive to ubiquitination-mediated proteasomal degradation by an E3 ubiquitin ligase (76–78). In addition, FIH-1 (Factor inhibiting HIF-1) also hydroxylate HIF-1α at asparagine residue, which further block the recruitment of p300/CBP therefore limiting the transcriptional activity of HIF-1α (79, 80). Under physiological hypoxia, the balance between nitric oxide and reactive oxygen species (ROS) were found to stabilize HIF-1α (81). The role of HIF-1α is quite established in Th cell differentiation and functions (82). HIF-1α promotes differentiation of Th17 cell by forming a tertiary complex with RORγt and p300 to *Il17* promoter, thus enhancing the transcription of IL-17 gene and targeting ubiquitination-mediated proteasomal degradation of Foxp3, resulting in reinforcing the development of Th17 while diminishing the generation of Tregs (83). Taken together these observations thus imply that transcription factor HIF-1α act as a metabolic checkpoint between Th17 and Tregs cell differentiation and functions (84). Consistently, these observations were further supported with the fact that HIF-1α-conditional deficient animals were found to be relatively resistant to the development of EAE associated with a reduced frequency of Th17 cells (84). In addition, the role of HIF-1α has been found to be associated with Th1 and Th2 cells; however, the deficiency of HIF-1α was not found to impair Th1 and Th2 cell differentiation, suggesting a dispensable role of HIF-1α in Th1 and Th2 cell differentiation (85). In addition, it has been recently reported that HIF-1α increases glycolytic activity in development of Th9 cells. Mechanistically, HIF-1α was found to induce IL-9 promoter activity by binding directly to *Il9* promoter in Th9 cells (17). Taken together these observations suggest that HIF-1α is critical for T cells activation and proliferation as well as for their differentiation into various effector T cells.

### IRF4

The transcription factor, interferon regulatory factor 4 (IRF4) is required for TCR-mediated metabolic programming in T cells (86). IRF4 expression is induced upon TCR stimulation, which was found to drive transcriptional program required for the differentiation of Th cell lineages. IRF4 translates TCR-affinity signals into the proliferation of appropriate T cell lineages, and is required for the survival of activated T cells (86). IRF4 binds to promoters of the genes and other downstream factors that are required for differentiation and key metabolic functions in T cells. Association of IRF4 with AP-1, c-Myc and HIF-1α regulates metabolic programming in activated T cells (86). Specifically in the Th differentiation, the initial role of IRF4 was found to be associated with Th2 differentiation, as IRF4-deficient T cells were failed to differentiate into Th2 cells (87). The role of IRF4 was further tested in other Th subsets such as Th17 and Th9 cells. Since Th2 and Th9 cells are sister populations as they share common differentiation factors, therefore, similar to Th2, the role of IRF4 was found to be crucial in Th9 development (88). IRF4 deficiency in animals leads to a defective Th9 cells development and associated with less severe allergic inflammation (88). The role of IRF4 was further identified in the development and functions of Th17 cells (89). TCR mediated induction of IRF4 is crucial for the induction of IL-21, which is found to be a crucial cytokine for amplification of Th17 cells (89). In addition to effector cell populations such as Th2, Th9, and Th17, the role of IRF4 was found to be associated with the function and development of Tregs and Tr1 cells. IRF4 deficiency in Foxp3^+^ Tregs leads to defect in Tregs function, which lead to Th2 cell-mediated tissue inflammation (90). It was further suggested that IRF4 in Tregs cells control Th2 mediated tissue pathology (90). In addition to Foxp3^+^ Tregs, the role of IRF4 has been also identified in Tr1 cells (91). Taken together these observations indicate that IRF4 is a critical metabolic checkpoint that is crucial for maintaining effector and regulatory T cells generation and functions.

### BCL-6

The transcriptional repressor B-cell lymphoma 6 (Bcl-6) play a role in metabolic regulation of T cells, and is essential in promoting the generation of memory T cells in both CD4^+^ and CD8^+^ compartments (92). In addition, the role of Bcl-6 has been demonstrated in shaping the development of T follicular helper (Tfh) cells (93), as the balance between Bcl-6 and Blimp1 was found to be critical in generation of Tfh cells, which are crucial for the formation of germinal centers (93).

As a repressor, Bcl-6 is found to downregulate genes of the glycolytic pathways by directly binding to their respective promoters (94). The repression of glycolysis by Bcl-6 is directly linked to IL-2 concentration, as higher concentration of IL-2 leads to downregulation of Bcl-6 and thereby upregulate glycolysis in proliferating T cells. Once the concentration of IL-2 limits, Bcl-6 gets upregulated, and therefore controls T cell proliferation by limiting the glycolysis and generation of metabolic precursors that are crucial for T cells growth (94).

In addition to controlling T cells proliferation, Bcl-6 was also found to influence Th cell differentiation. Bcl-6 was found to inhibit Th1 and CD8^+^ Tc1 cell differentiation by repressing glycolysis which is the major metabolic pathway in these T cell subsets (94). Interestingly, T-bet, a master transcription factor of Th1 lineage, is reported to inhibit Bcl-6, and overcome the repression of glycolysis by Bcl-6 in Th1 cells (94). The Bcl-6 mediated repression of glycolytic gene program in effector T cells is so dominant that it cannot be restrained by HIF-1α and c-Myc, which are also known to upregulate glycolysis (94). Bcl-6 by inhibiting glycolysis creates a switch from effector to memory T cell and thus promotes memory cell formation. The role of Bcl-6 was also found to be associated in Th9 cells, as Bcl-6 is found to impair Th9 differentiation by competing with STAT5/STAT6 binding sites on *Il9* promoter, and thus represses the transcription of *Il9* gene (95). IL-2 induces Th9 differentiation while IL-21 inhibits Th9 differentiation by differential regulation of Bcl-6 expression (95, 96). These observations suggest that Bcl-6 act as an important metabolic regulator of Th differentiation pathways.

### Foxo

Foxo1 is an also essential metabolic checkpoint transcription factor in Th differentiation. Forkhead box O (Foxo) family of transcription factors consists of four Foxo family members: Foxo1, Foxo3, Foxo4, and Foxo6. Foxo1 and Foxo3a were found to be most abundant in T cells, therefore the role of Foxo1 and Foxo3a was extensively studied in T cell development, differentiation and functions (97). Foxo1 and Foxo3 plays a critical role in cell metabolism, apoptosis, cell cycle progression, and detoxification of reactive oxygen species (98, 99). The DNA binding motif of Foxo has been characterized and found to be conserved. The role of Foxo1 and Foxo3 was widely studied in both iTregs and nTreg cell development and functions, as mice deficient in both Foxo1 and Foxo3 have decreased number of nTregs with loss of their suppressive functions (100). Similarly, Foxo1 and Foxo3 deficient mice showed impaired generation of induced Treg cells (Foxp3^+−^iTregs), which is induced in the presence of TGF-β1 (100). Consistent with these observations, Foxo1, and/or Foxo3a deficient mice develop severe colitis due to non-functional Tregs (100). In addition to Tregs, the role of Foxo1 was found to be associated with Th17 cells differentiation and function, as Foxo1 deficient mice were found to be susceptible to EAE associated with an enhanced frequency of Th17 cells (101). In addition, it was shown that Foxo1 suppressed the functions of RORγt by directly binding to it, and therefore leads to decreased Th17 cells generation (101). It was further identified that IL-23-IL-23R pathways regulated Foxo1 functions via SGK1, which induces the generation of pathogenic Th17 cells (102).

It has been demonstrated that AKT phosphorylates Foxo at Ser^253^ residue causing its nuclear exclusion and ubiquitin mediated degradation. The inhibition of PI3K-AKT pathway promotes Treg cell differentiation through the activation of Foxo (100). Naïve Foxo1-deficient CD4^+^ T cells become T-bet^+^IFNγ^+^Th1 cells and fail to differentiate into Foxp3^+^ Treg cells (103). TGF-β-induced Foxo1 inhibits the expression of T-bet while the activation of S1P1 signaling interferes with TGFβ-SMAD3 pathway by activating S6 kinases, downstream of S1P1-induced activation of mTORC1 pathway. This inactivates Foxo1 and thus potentiates Th1 cell differentiation (103). Recent data from our group has identified the crucial role of Foxo1 in promoting Th9 cell differentiation, through inhibition of PI3K/AKT pathway, which activates Foxo1 and increases IL-9 production in Th2, Th9, and Th17 cells. Loss of Foxo1 attenuates IL-9 in Th9 cells and ameliorates allergic inflammation in asthma (104). Moreover, the activation of STAT5 and PI3K-AKT-mTOR signaling pathway by IL-7 increases the abundance of the histone acetyl transferase p300 which promotes the acetylation of histones at the *Il9* promoter resulting in dephosphorylation of Foxo1 thus inducing the production of IL-9 and potentiating Th9 cell differentiation (105). Deficiency of Foxo1 inhibits IL-7 mediated Th9 cell differentiation and antitumor activity of Th9 cells (105). These observations clearly indicate that Foxo proteins are critical metabolic checkpoints that affect T cells differentiation and functions in immunity and autoimmunity.

### PPARγ

Peroxisome proliferator-activated receptor gamma (PPARγ) is a ligand-dependent transcription factor which is known to regulate glucose and lipid metabolism, cell growth, differentiation and apoptosis. PPARγ-deficient T cells display enhanced proliferation with an increased activation of ERK and AKT, which lead to increased cytokine production under Th1, Th2, Th9, and Th17 differentiation conditions (106). PPARγ increases the stability of IκBα, Foxo1, and Sirt1, which are the negative regulators of NF-κB, thus inhibiting T cell proliferation (106). In addition to T cell proliferation, the role of PPARγ was also found to be associated with T cells activation and differentiation. The role of PPARγ was shown in the differentiation of Th17 cells, as PPARγ was found to interfere with TGF-β/IL-6-induced transcriptional activation of RORγt by preventing the removal of corepressor from the RORγt promoter and thus regulate Th17 cells differentiation. Consistently, PPARγ deficient mice were found to be severely susceptible for tissue inflammation in EAE due to an increased frequency of Th17 cell (107). However, PPARγ activation in CD4^+^ T cells didn't show any inhibitory effect on Th1, Th2, or Treg cell differentiation (107).

## Signaling Transducers and Sensors as Metabolic Checkpoints of T cell differentiation

### mTOR

The mTOR mammalian target of rapamycin (mTOR) is an evolutionary conserved serine/threonine kinase that is a part of PI3K-AKT pathway. mTOR comprises of two functionally distinct complexes: mTORC1 and mTORC2, which play distinct role in Th differentiation. mTORC1 is composed of regulatory associated protein of mTOR (Raptor), Rheb (a small GTPase), G protein β-subunit-like protein (GβL), mammalian lethal with Sec13 protein 8 (mLST8), the proline-rich Protein Kinase B (PKB)/Akt substrate of 40 kDa (PRAS40), and DEP-domain-containing mTOR-interacting protein (DEPTOR). On the other hand, mTORC2 comprises of mLST8 and DEPTOR, rapamycin-insensitive companion of TOR (RICTOR), mSIN1 (mammalian stress-activated protein kinase interacting protein-1) and the protein observed with RICTOR (PROTOR) (108–110). Both mTORC1 and mTORC2 was found to have differential sensitivity for rapamycin, as mTORC1 is rapamycin sensitive and plays a role in autophagy, protein translation, and ribosome biogenesis while mTORC2 is rapamycin insensitive. mTORC1 activation leads to phosphorylation and activation of the ribosomal S6 kinase 1 (S6K1) while mTORC2 phosphorylates Akt at serine 473 (111). mTOR is crucial for regulating glucose metabolism and upregulates glycolysis in T cells, as mTOR–/– cells were shown to have decreased glycolytic activity. The mTOR pathways were found to play an essential role in T cells differentiation and functions. mTOR is required for effector T cell lineage commitment, as activated T cells lacking mTOR fails to differentiate into effector T cell and rather they differentiate into Foxp3^+^ Tregs (111). Mechanistically, lack of mTOR leads to Smad3 activation even in absence of TGF-β1 which potentiate the generation of Foxp3^+^ Tregs. The role of mTORC1 and mTORC2 was found to be associated with Th1 and Th2 differentiation, respectively. CD4^+^ T cells lacking mTORC1 activity were found to have reduced phosphorylation of STAT4 in response to IL-12, which leads to reduced generation of Th1 cells. Although mTORC1 activity was found to be indispensable for Th1 cell differentiation, Th2 cells differentiate even in absence of mTORC1 but they require mTORC2 activity for their differentiation (111). Like Th1 cells, mTORC1 was found to be essential for the differentiation of Th17 cells while deficiency of mTORC2 was not found to be associated with Th17 cells differentiation (111). Recently it has been reported that mTOR induces Th9 cell differentiation by upregulating glycolytic pathway in HIF-1α dependent manner (17). mTORC1 regulates glucose metabolism and function in CD8^+^ T cell independent of PI3K and PKB (108).

### AMPK and LKB1

Activated T cells possess AMP-activated protein kinase (AMPK), a glucose-sensitive metabolic checkpoint, found to regulate mRNA translation and glutamine-dependent mitochondrial metabolism. AMPK is a heterotrimeric serine/threonine kinase complex which promotes energy conservation in T cells and primarily involved in maintaining T cell bioenergetics and viability (112, 113). During prolonged starvation and stress conditions, AMPK promotes catabolic processes like fatty acid oxidation for limiting energy expenditure and replenishing ATP production rather than anabolic processes which consume ATP (112, 113). AMPK promotes fatty acid oxidation by increasing the expression and phosphorylation of carnitine palmitoyl transferase 1A (CPT1A), which is the rate-limiting enzyme and inhibits acetyl-CoA carboxylase 2 (ACC2) (114). AMPK also enhances mitochondrial biogenesis by promoting the transcriptional activity of peroxisome proliferator-activated receptor-γ coactivator 1α (PGC1α) and oxidative metabolism (114). Furthermore, AMPK found to inhibit glycolysis, glutaminolysis, glycogen and fatty acid synthesis while it promotes oxidative phosphorylation and autophagy (112–114). AMPK is activated by phosphorylation of α subunit at Thr172 by LKB1 under the conditions of bioenergetic stress (113). AMPK is also activated via Ca^2+^-calmodulin-dependent protein kinases upon TCR triggering (20). AMPK inhibits mTORC1 activity by phosphorylation of TSC2 and RAPTOR, which are important for its activity, thus inhibiting the T helper cell differentiation. T cells deficient in AMPKα1 found to display reduced mitochondrial bioenergetics and metabolic plasticity in response to glucose limitation (112–114). Glucose limitation affect Th1 differentiation by reducing the mRNA and protein level of IFN-γ (115). AMPK play a major role in limiting IFN-γ production under glucose unavailability. AMPKα1-deficient T cells produced enhanced IFN-γ even in the metabolically unfavorable condition (115). As discussed above that effector Th cells relies on glycolysis while Tregs requires on FAO. AMPK is highly enriched and active in Tregs, as it was found that AMPK drives naïve T cells into Tregs (14, 29, 116). AMPK found to negatively regulate glycolysis, which is required for the generation of effector Th subsets. Consistently, metformin, an activator of AMPK, found to block Th17 cell differentiation *in vitro* and *in vivo* and alters the ratio of Th17: Tregs in mouse model of colitis and asthma (117, 118). Consistently, metformin treatment was found to ameliorate tissue inflammation in mouse models of autoimmune diseases. Taken together, it indicates that AMPK is crucial for maintaining the balance between effector and regulatory T cells.

The Liver Kinase B1 (LKB1) is a serine/threonine kinase that links cellular metabolism with cell growth and proliferation (119). LKB1 was initially identified in Peutz-Jeghers syndrome, an autosomal dominant disorder that leads to carcinoma (119). LKB1 was found to be upstream of AMPK and two together regulate metabolically active T cells. In fact, LKB deficiency leads to decreased activation of AMPK. LKB1 is a key regulator of lipid and glucose metabolism in T cells where loss of LKB1 increases glucose metabolism (120). Similar to AMPK, LKB1 regulates the functions of both CD4^+^ and CD8^+^ T cells during inflammation (121, 121). LKB1 primarily regulates metabolism in T cells via AMPK-dependent and independent pathways. In fact, a subset of LKB1 functions was found to be carried out by AMPK, as deletion of AMPK display similar defect in T cell activation. T cell-specific deletion of the gene that encode LKB1 found to affect the number of thymocytes and peripheral T cells (121). In addition, LKB1 deficient T cells were shown to have enhanced activation and cytokine production, which could be due to altered glycolytic and lipid metabolism (121). Loss of LKB1 promotes Th1 and Th17 cell differentiation. AMPK and LKB1 together negatively regulate the inflammatory cytokine production by T cells by inhibiting mTORC1 signaling, which is critical for inflammatory cytokine production (121). These observations indicate the role of LKB1-AMPK axis in metabolism of T cells affecting their differentiation and functions.

### SIRT1

SIRT1 is a mammalian homolog yeast NAD^+^ dependent type III histone deacetylase which plays an important role in a variety of biological and metabolic processes in immune cells. SIRT1 is highly expressed in dendritic cells (DC) and reciprocally regulates Th1 and iTreg cell differentiation (122). SIRT1 signaling in DCs inhibits Th1 differentiation while promotes Tregs generation via modulation of DC derived T-cell polarizing cytokines such as IL-12 and TGF-β1 in a HIF-1α dependent manner (122). SIRT1-HIF1α signaling axis in DC reciprocally regulates Th1 and iTreg cell generation by modulating IL-12-STAT4 and TGFβ1-SMAD3 pathways in a mTOR independent manner (122). However, an unexpected proinflammatory role of SIRT1 has been established in the generation and functions of Th17 cells. SIRT1 activates RORγt by deacetylation enhancing Th17 cell differentiation thereby promoting autoimmunity (123). In addition to Th1, Th17, and Tregs, the role of SIRT1 has also been established in the differentiation and functions of IL-9-producing Th9 cells (17). SIRT1 inhibits Th9 cell differentiation by negatively regulating mTOR-HIF1α dependent glycolytic activity (17). Overexpression of SIRT1 negatively regulates IL-9 producing CD4^+^ T cell differentiation while SIRT1 deficiency promotes Th9 cell differentiation by inducing mTOR-HIF1α dependent glycolytic activity (17). Taken together observations clearly indicate that SIRT1 is one of the crucial metabolic checkpoints in regulating T cells differentiation and functions in tissue inflammation.

## Metabolic Checkpoints During Tissue Inflammation

T cell encounters a variety of micro-environmental cues during differentiation into effector and regulatory T cells. The molecules which are essential for host metabolism affect and modulate T cells functions. In addition to antigen and cytokines, metabolic precursors, play a key role in activation, proliferation, and differentiation of T cells that lead to diverse immunological responses in tissue inflammation. An activated effector T cell undergoes a dramatic metabolic shift to support its growth, proliferation, differentiation, and functions. This metabolic shift occurs during T cells activation in tissue inflammation due to limited availability of oxygen and nutrients. Tumor infiltrating lymphocytes (TILs) are good example of such changes, where tumor microenvironment support less oxygen and glucose, which leads to metabolic shift in TILs (124–126). Under hypoxic environment in tumor, T cells express HIF-1α, which further modulate the course of immune response in tumors and its associated inflammation (127).

The metabolic shift known as Warburg effect is an essential phenomenon of active and growing T cells in tissue inflammation to support its energy demand in terms of ATP production and synthesis of biosynthetic precursor and intermediate molecules (128). The Warburg effect is particularly evident during tissue inflammation when activated T cell shifts toward aerobic glycolysis providing glucose-6-phosphate for the pentose phosphate pathway (PPP) generating 3-phosphoglycerate. This 3-phosphoglycerate is then utilized for the serine biosynthetic pathway which is essential for the biosynthesis of various cytokines required to induce effector functions of T cells in tissue inflammation. The aerobic glycolysis provides pyruvate to the TCA cycle leading to the synthesis of citrate, which is required for the membrane fatty acid synthesis (129). Metabolic reprogramming of T cells in tissue inflammation toward aerobic glycolysis further allow these cells to overcome stressful microenvironment, such as reduced cellular oxygen level, during tissue inflammation (130, 131). Moreover, lesser amount of ATP is generated through oxidative phosphorylation due to low levels of oxygen in the vicinity of tissues during inflammation (132, 133). Thus, aerobic glycolysis constitutes the major metabolic pathway in activated T cells, activated B cells, activated macrophages, DCs, stimulated natural killer cells and neutrophils during tissue inflammation (2, 9, 84, 134–139).

The metabolic reprogramming of T cells is well controlled at various metabolic checkpoints during homeostasis and inflammation. Metabolic shift toward aerobic glycolysis is supported by an enhanced expression of GLUT1 for increased glucose transport inside the cell for enhancing the rate of aerobic glycolysis (12). Interestingly, effector T cells efficiently adapts to hypoglycemia at the site of inflammation where glucose levels are low. They do so by internalizing glutamine from their surrounding environment and catabolizes it through glutaminolysis for the continuity of TCA cycle (136). The glutamine supply to the differentiating effector T cells is crucial for maintaining Th1 cell differentiation, as it is observed that in the absence of glutamine during tissue inflammation, T cell skew toward Treg phenotype (140–142). Recently, similar role of amino acids such as leucine and arginine has also been established for differentiation and functions of effector T cells (143, 144).

The key players that bring about these metabolic reprogramming are mTOR and transcription factors such as Myc and HIF-1α as discussed above. Activity of these regulators in turn modulates the AMPK activity (145). mTOR activation is responsible for a number of dynamic changes within the proliferating activated T cells such as enhanced mRNA translation and fatty acid synthesis, maintenance of compartment Myc levels which in turn, is crucial for the induction of glycolytic gene expression (73, 143, 146, 147). Remarkably, different mTOR complexes triggers distinct metabolic programming that lead to effector vs. memory T cell generation (134). For example, mTORC1 signaling shifts the metabolism toward aerobic glycolysis during the proliferation of effector T cells; whereas mTORC2 is required for the metabolic reprogramming in memory T cells (134). Reduction of glycolysis together with an increased oxidative catabolism downregulate mTOR signaling, which skew T cells to become Tregs. Taken together it clearly indicates that mTOR serves as a key metabolic checkpoint for the development of effector and regulatory T cells that influences the outcome of tissue inflammation (111). The role of AMPK was also suggested in modulating effector and regulatory T cell response, as AMPK were shown to promote the development of regulatory and anti-inflammatory T cells while limiting the generation of effector T cells (145). Low cellular energy and insufficient nutrient supply trigger AMPK activation leading to inhibition of mTOR and upregulation of fatty acid catabolism, which support Tregs development and functions. In addition, at transcriptional level, upregulation of HIF-1α and Myc gene expression along with suppression of Bcl-6 is essential for the metabolic reprogramming in T cells during tissue inflammation (73, 84, 148, 149), which is initiated by Myc in CD4^+^ T cells while it is maintained further in CD8^+^ T cells by AP-4 and IRF4 transcription factors as soon as Myc activity declines (86, 150). Thus, these metabolic checkpoints play a crucial role in tissue inflammation and have enormous potential for immunotherapy.

## Conclusion

The metabolic checkpoints are essential to maintain balance between pro- and anti-inflammatory T cells during tissue inflammation. These metabolic checkpoints include various cellular components ranging from cellular metabolites, cell signaling molecules and transcription factors. The dynamic interactions between these checkpoints determine the outcome of T cell response in tissue inflammation. Emerging data clearly indicate that metabolic checkpoints are different for effector, memory, and regulatory T cells, and so does the factors that regulate these metabolic reprogramming. As discussed above in this review, a dysregulated metabolic checkpoint is indicative of imbalance between effector, memory, and regulatory T cells. Many of these metabolic checkpoints are in an area of active research and being tested as potential therapeutic targets for inflammatory diseases. However, the regulatory metabolic network of these checkpoints in tissue inflammation, autoimmune diseases as well as in infection is not completely understood. Nonetheless, several therapeutic drugs are available or undergoing clinical trials for ameliorating tissue inflammation targeting metabolic checkpoints as summarized in Table 1. More broader understanding is required for different metabolic checkpoints to formulate new immunotherapies as well as immunomodulation for various inflammatory conditions and tumor microenvironment.

**Table 1:** List of drugs targeting metabolic checkpoints in clinical trials in inflammation and cancer.

**Metabolic checkpoints**	**Drugs**	**Disease/Condition**	**Mechanism of action**
Glucose	Glitazones	Diabetes Mellitus (Type 2), Inflammatory Bowel Disease (IBD), Severe Coronary Artery Disease, Obesity.	PPARγ agonist leading to increased glucose metabolism and lower free fatty acid in circulation
	Glucophage	Diabetes Mellitus (Type 2), Stable Coronary Artery Disease, Obesity, Impaired Glucose Tolerance	Lowers liver glucose production. Putative action mode is through activation of AMPK or inhibition of cAMP, etc.
Fatty Acids	Omegavan	Crohn Disease (CD)	Largely unknown, the effect is believed to be through modulation in free fatty acid constitution.
	Eicosa-pentaenoic acid	Ulcerative Colitis (UC), CD	It is a precursor for bio-active lipids such as leukotriene-5 eicosanoids, thromboxane-3 and prostaglandin-3.
	Epanova	CD	It results in low triglyceride burden by limiting its production. The mechanism though not completely understood appears to be mediated through fatty acid metabolism and synthesis.
	Intralipid	CD	Intralipid formulation provides fatty acids essential for the body. The effects appear to be mediated through omega-3 and omega-6 fatty acids and also through linoleic acid.
Cholesterol	Rosuvastatin	Carotid Artery Plaque, Ankylosing Spondylitis, Rheumatoid Arthritis (RA), Chronic Obstructive Pulmonary Disease (COPD), CD	It acts by modulating the lipid profile in patients It acts as a competitive inhibitor for HMG-CoA reductase.
	Atorvastatin	Chronic Kidney Disease, CD, RA, Non-Cystic Fibrosis Bronchiectasis	It acts by modulating the lipid profile in patients It acts as a competitive inhibitor for HMG-CoA reductase.
	Simvastatin	Carotid Artery Disease (CAD), COPD, Atherosclerosis, Cardiovascular Disease	It acts by modulating the lipid profile in patients It acts as a competitive inhibitor for HMG-CoA
	Flax seed	Non Alcoholic Steatohepatitis, Cardiovascular Disease	Exact mechanism is unknown; however it is known to change the lipid profile.
	GSK2982772	IBD	It is a receptor-interacting protein-1 (RIP1) kinase inhibitor
	Niacin	Dyslipidemia, Atherosclerosis, Cardiovascular Disease	It act as a precursor for nicotinamide which is required for many enzyme complexes.
	Vitamin E	COPD, CD, UC, Chronic Kidney Disease	It is known as a fat soluble antioxidant. It has peroxyl radical scavenging activity. Its role is to prevent oxidation of fatty acids.
Nitric Oxide	Sapropterin	Pulmonary Disease, Chronic Obstructive	It is a cofactor component of 3 aromatic amino acid hydroxylase enzymes.
	Pyridoxalated haemoglobin polyoxyethylene conjugate (PHP)	Systemic Inflammatory Response Syndrome	It acts by modulating the lipid profile in patients It acts as a competitive inhibitor for HMG-CoA
ATP	Mesalazine	UC, CD	Not completely understood
	Methotrexate	UC, CD, RA, Psoriasis	It acts as an antifolate which means it inhibits folate that is essential for purine and pyrimidine synthesis. As a result methotrexate inhibits DNA, RNA, thymidylates and protein synthesis.
	Azathioprine	UC, CD, RA	It inhibits purine synthesis It acts as an immunosuppressant as it inhibits DNA, RNA synthesis.
	Allopurinol	IBD	It is a xanthine oxidase inhibitor which decreases blood uric acid levels
	Prednisone	IBD	Prednisone acts as a corticosteroid that regulates glucose levels and is effectively used as an immunosuppressant drug.
HIF-1α	EZN 2968	Neoplasms, Lymphoma, Carcinoma	It is an inhibitor of HIF-1α transcription factor.
	Doxorubicin	Inflammatory Breast Cancer	It inhibits topoisomerase II activity by intercalating with DNA
	Docetaxel	Inflammatory Breast Cancer	It disrupts functional microtubules thus inhibiting cell division
	Cyclo-phosphamide	Inflammatory Breast Cancer, Vasculitis	It is an alkylating agent and therefore inhibits DNA replication and transcription of RNA
	Vildagliptin	Diabetes Mellitus Type 2, Ischemic Heart Disease	It acts as an anti-hyperglycemic agent by inhibiting the inactivation of Gastric inhibitory polypeptide (GIP) glucagon-like peptide-1 (GLP-1).
	RAD001	Coronary Artery Disease, CD	It is an inhibitor of mammalian target of rapamycin (mTOR) with more inhibitory effect for mTORC1 as compared to mTORC2. It is used as an immunosuppressant.
BCL-6	Rituximab	Behcet's Syndrome Arthritis, Rheumatoid Spondyloarthritis	It is an antibody against CD20 protine on B cells. Its binding leads to Ca2^+^ influx, resulting in B cell activation. Further, it is also known to enhance MHC II molecule while downregulating BCR.
FOXO	Resveratrol	Coronary Artery Disease	Mechanism not fully understood. *In vitro* it acts as an activator of sirtuin 1 and modulates mitochondrial functioning.
PPARγ	Pioglitazone	Type 2 Diabetes Mellitus, Thyroid Cancers	It induces hypoglycemia by activating PPAR-γ and PPAR-α (to a lesser extent). Furthermore, it also regulates the transcription of glucose and lipid metabolism genes in liver, adipose tissues and muscles, thus regulating the blood glucose level.
	Rosiglitazone	Type 2 Diabetes Mellitus, Hypertriglyceridemia in Type 4 Hyperlipidemia	It acts by binding to PPARs thereby modulating the expression of genes involved in glucose and fatty acid metabolism. Apart from its anti-insulin nature, rosiglitazone are known to posses anti-inflammatory properties as well by down-regulating NF-κB levels.
	GW 501516	Hypercholesterolemia, Dyslipidaemias, Obesity	It is a synthetic PPAR receptor agonist which specifically recognizes PPARδ receptor agonist. In laboratory animal models GW 501516 has been shown to increase fatty acid metabolism and protect against type II diabetes and obesity.
	Fenofibrate	Hypertension, Dyslipidemia, Hypertriglyceridemia, Insulin Resistance	It activates PPARα and other PPAR family which in turn activates various enzymes and signaling mediators to promote cholesterol and triglyceride metabolism.
mTOR	Tacrolimus	Hepatocellular Carcinoma, CD	It inhibits T cell TCR activation and T cell proliferation. The mechanism for this inhibition is through calcineurine inhibition, a calcium sensing molecule that is essential for NFAT activation and IL-2 secretion.
	Rapamycin	Vascular Malformations, Neurofibromas,	It is an inhibitor of mTOR and therefore it interferes with T cell and B cell activation. It inhibits IL-2 and therefore act as an immune-suppressant for humans.
	BEZ235	Malignant Solid Tumor, Pancreatic Neuroendocrine Tumors (pNET),	It is a known inhibitor of mTOR. It also inhibits PI3K activity.
LKB1	Erlotinib	Non-Small Cell Lung Cancer	Erlotinib is a potent EGFR and JAK2V617F inhibitor.
	AZD6244	Non-Small Cell Lung Cancer, Skin Melanoma	The mechanism of Selumetinib action is not completely understood. Much of its inhibitory effect is believed to be because of blocking MEK1 and MEK2 enzyme complex.
SIRT1	Melatonin	Multiple Sclerosis, Oxidative Stress	It is a hormone produced by pineal gland in humans. How melatonin influences SIRT1 is not well understood, however, many of its physiological effects are because of melatonin receptor activation and antioxidant property of melatonin.
	SRT2379	Endotoxin-Induced Inflammation, Sepsis, Diabetes Mellitus, Type 2	It is a synthetic molecule which is known to activate SIRT1. The mechanism of this activation however, remains largely unelucidated.

## Author Contributions

SR, ZAR and AA contributed to writing the review. AA and SR corrected and edited the review.

### Conflict of Interest Statement

The authors declare that the research was conducted in the absence of any commercial or financial relationships that could be construed as a potential conflict of interest.
